# A new HaCV-EBHSV recombinant lagovirus circulating in European brown hares (*Lepus europaeus*) from Catalonia, Spain

**DOI:** 10.1038/s41598-024-53201-1

**Published:** 2024-02-04

**Authors:** Tereza Almeida, Ana M. Lopes, Josep Estruch, Carlos Rouco, Patrizia Cavadini, Aleksija Neimanis, Dolores Gavier-Widén, Ghislaine Le Gall-Reculé, Roser Velarde, Joana Abrantes

**Affiliations:** 1 CIBIO, Centro de Investigação em Biodiversidade e Recursos Genéticos, InBIO Laboratório Associado, Campus de Vairão, Universidade do Porto, 4485-661 Vairão, Portugal; 2 BIOPOLIS Program in Genomics, Biodiversity and Land Planning, CIBIO, Campus de Vairão, 4485-661 Vairão, Portugal; 3 UMIB-Unit for Multidisciplinary Research in Biomedicine, ICBAS-School of Medicine and Biomedical Sciences, University of Porto, Porto, Portugal; 4 ITR, Laboratory for Integrative and Translational Research in Population Health, Porto, Portugal; 5 Wildlife Ecology & Health Group (WE&H) and Servei d’Ecopatologia de Fauna Salvatge (SEFaS), Departament de Medicina i Cirurgia Animals, Universitat Autònoma de Barcelona (UAB), Barcelona, Spain; 6 Departamento de Biología Vegetal y Ecología, Universidad de Sevilla, Sevilla, Spain; 7 WOAH Reference Laboratory for Rabbit Haemorrhagic Disease, Istituto Zooprofilattico Sperimentale della Lombardia e dell’Emilia Romagna, Brescia, Italy; 8 Department of Pathology and Wildlife Diseases, National Veterinary Institute, 75189 Uppsala, Sweden; 9 Swedish Veterinary Agency (SVA), 75189 Uppsala, Sweden; 10 Department of Biomedical Sciences and Veterinary Public Health, Swedish University of Agricultural Sciences (SLU), Box 7028, 75007 Uppsala, Sweden; 11 Avian and Rabbit Virology, Immunology and Parasitology Unit, Ploufragan-Plouzané-Niort Laboratory, French Agency for Food, Environmental and Occupational Health & Safety (Anses), 22440 Ploufragan, France; 12 Departamento de Biologia, Faculdade de Ciências, Universidade do Porto, 4099-002 Porto, Portugal

**Keywords:** Molecular evolution, Virology

## Abstract

In 2020/2021, several European brown hare syndrome virus (EBHSV) outbreaks were recorded in European hares (*Lepus europaeus*) from Catalonia, Spain. Recombination analysis combined with phylogenetic reconstruction and estimation of genetic distances of the complete coding sequences revealed that 5 strains were recombinants. The recombination breakpoint is located within the non-structural protein 2C-like RNA helicase (nucleotide position ~ 1889). For the genomic fragment upstream of the breakpoint, a non-pathogenic EBHSV-related strain (hare calicivirus, HaCV; GII.2) was the most closely related sequence; for the rest of the genome, the most similar strains were the European brown hare syndrome virus (EBHSV) strains recovered from the same 2020/2021 outbreaks, suggesting a recent origin. While the functional impact of the atypical recombination breakpoint remains undetermined, the novel recombinant strain was detected in different European brown hare populations from Catalonia, located 20–100 km apart, and seems to have caused a fatal disease both in juvenile and adult animals, confirming its viability and ability to spread and establish infection. This is the first report of a recombination event involving HaCV and EBHSV and, despite the recombination with a non-pathogenic strain, it appears to be associated with mortality in European brown hares, which warrants close monitoring.

## Introduction

Lagoviruses (*Caliciviridae* family) include highly lethal RNA viruses with hepatic tropism that cause disease outbreaks in leporids (rabbits and hares) worldwide. Upon infection, significant morbidity and mortality rates are observed. The European rabbit (*Oryctolagus cuniculus*) is affected by rabbit hemorrhagic disease virus (RHDV; *Lagovirus europaeus*/GI, according to the nomenclature proposed by Le Pendu et al.^1^), which causes rabbit hemorrhagic disease (RHD). European brown hares and, to a lesser extent, mountain and Italian hares (*Lepus europaeus*, *L. timidus*, and *L. corsicanus*, respectively) are also infected by the European brown hare syndrome virus (EBHSV; *Lagovirus europaeus*/GII.1) causing the European brown hare syndrome (EBHS). Despite resulting in similar clinical signs and lesions, including hepatic necrosis, splenomegaly, and hemorrhages in multiple organs, RHDV and EBHSV differ, on average, ~ 30% in their nucleotide (nt) sequences, and have distinct antigenic profiles^2,3^.

**Figure 1:**
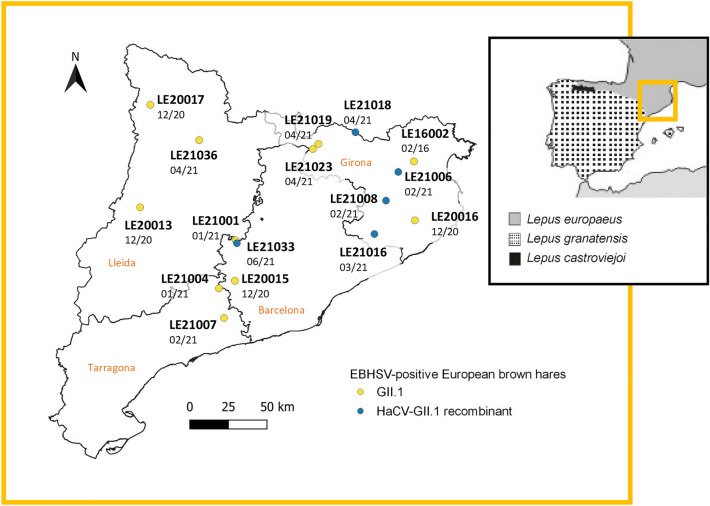
Geographical distribution of the European brown hare samples. Samples were collected in the four provinces of Catalonia, NE of Spain: Barcelona, Girona, Lleida and Tarragona. The samples codes and the date of collection (month/year) are indicated. Yellow dots refer to EBHSV (GII.1) strains, whilst blue dots correspond to HaCV-EBHSV (GII.1) recombinant strains. The map was created with Quantum GIS software 3.16 version (https://qgis.org/es/site/index.html). Hare species distribution in the Iberian Peninsula, including the sampling area (yellow square), is also shown: European brown hare, *L. europaeus* (grey), broom hare, *L. castroviejoi* (black), and Iberian hare, *L. granatensis* (dotted) (adapted from^52^ and reproduced with permission from the publisher John Wiley and Sons).

EBHS was first reported in Sweden in 1980, followed by several European countries, including Denmark, Belgium, England, Italy, France, Poland, Slovakia, Greece, Switzerland and Germany^4–13^, reflecting the distribution ranges of European brown hares (*L. europaeus*) and mountain hares (*L. timidus)*^14^. In the Iberian Peninsula, despite surveillance efforts, only two isolated cases of EBHSV have been reported in European brown hares within the restricted distribution of the species in this area^15^ (Fig. 1): one in Aragon (Central Pyrenees) in 1998^16^, and a second in 2016 in Catalonia (Oriental Pyrenees)^17^. No cases have been reported in other hare species inhabiting the Iberian Peninsula, such as the endemic Iberian hare (*L. granatensis*) and the broom hare (*L. castroviejoi*; Fig. 1)^15^, and, until recently, it was considered an EBHS-free region^18^.

Non-pathogenic EBHSV-related viruses also circulate in hares from Italy, Austria, Germany, France and Australia, and have been collectively named hare caliciviruses (HaCV; *Lagovirus europaeus*/GII.2 and GII.3; some HaCV strains are yet to be classified, but likely represent new genotypes)^19–21^. Similarly to non-pathogenic RHDV-related lagoviruses, i.e., GI.3 and GI.4, HaCVs have an enteric tropism^19–21^. These strains are genetically diverse, suggesting that they belong to different genotypes and have been circulating for some time^19,21,22^. Indeed, it was estimated that the time to the most recent common ancestor of HaCV GII.2 and GII.3 is 1879 (95% HPD: 1824–1922) and 1923 (95% HPD: 1876–1964), respectively^22^. While their role in EBHS epidemiology is unknown, the prevalence in hare populations ranges between 2.9 and 45%^19,21,22^. HaCV presence has been confirmed in European brown hares and in a single Corsican hare (*L. corsicanus*)^21^. In the Iberian Peninsula, HaCV has not been detected in the endemic Iberian hare^21^, but data suggests its circulation in European brown hares^18^.

**Figure 2:**
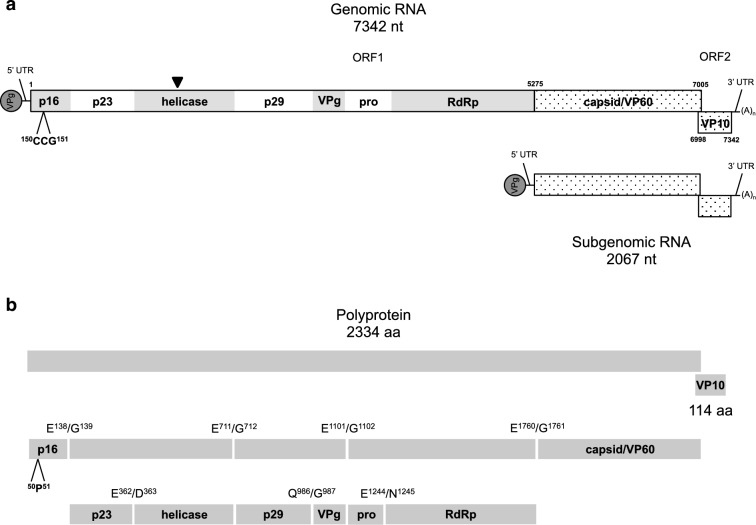
Schematic representation of open reading frames (*ORF*), gene order and polyprotein cleavage map of EBHSV according to the reference sequence EBHSV-GD (GenBank accession number: Z69620). (**a**) EBHSV virions have a genomic and a subgenomic RNA, which present short 5′ and 3′ untranslated regions (UTRs), are VPg linked and polyadenylated. Positions of *ORF1* and *ORF2* and of the subgenomic RNA transcription start site are indicated. The regions encoding the structural proteins VP60 and VP10 appear as dotted. The 3-nucleotide insertion is indicated. The arrowhead points to the location of the breakpoint of the reported recombinant strains. (**b**) *ORF1* encodes a polyprotein that is post-translationally cleaved into several proteins. The position of the cleavage sites and the products are indicated. The amino acid insertion is indicated.

In lagoviruses, the genome is organized into two open reading frames (*ORFs*). *ORF1* encodes a polyprotein which, upon maturation, originates seven non-structural proteins, p16, p23, 2C-like RNA helicase, p29, VPg, 3C-like protease, and RNA-dependent RNA polymerase (RdRp), and the major structural protein, VP60 or capsid; *ORF2* encodes a minor structural protein, VP10 (Fig. 2). Recombination is an important mechanism to generate genetic diversity in lagoviruses and several events have been described with a breakpoint close to the boundary between the polymerase and the capsid protein, being considered a recombination hotspot^19,23–32^. Other recombination breakpoints have been reported scattered throughout the genome^23,29,33,34^. Moreover, recombination might be linked to the emergence of GI.1 and GI.2 genotypes as pathogenic strains^23,26,35^, confirming its importance in the evolution of lagoviruses. Regarding genotype GII, fewer events have been described, but recombination was recently reported between GII.1 and GI.2 (GII.1P-GI.2)^31^ and between HaCV strains^19^.

From December 2020 to June 2021, unusual outbreaks of EBHS were reported in European brown hares from Catalonia, Spain^17^. In this study, we performed a genomic characterization of the causative viral strains and show the emergence of a novel and viable recombination event between HaCV and GII.1. Due to the associated mortality, further monitoring is advised.

## Results

### Characterization of the complete coding sequences

We obtained the complete coding sequences of 16 lagoviruses detected in the same number of European brown hares found dead in the field in the Spanish provinces of Barcelona, Lleida, Girona and Tarragona, in 2016 and between 2020 and 2021. The coding sequences of 11 viruses were 7342 nucleotides (nt) in length, while the remaining five were 7345 nt. Sequence comparison to the reference strain EBHSV-GD (GenBank accession number Z69620) showed the typical features of *Lagovirus europaeus*/GII, with two *ORFs* (Fig. 2a). *ORF1* was 7005 or 7008 nt long and encoded a protein with 2334 or 2335 amino acids (aa). *ORF2* (nt positions 6998–7342) was 345 nt long and encoded a 114 aa long protein. Translation of *ORF1* and its corresponding protein sequence alignment with those from other lagoviruses revealed a conserved organization of the nonstructural proteins (p16, p23, 2C-like RNA helicase, p29, VPg, 3C-like protease, and RNA-dependent RNA polymerase (RdRp)). *ORF2* seemed to encode the minor structural protein VP10. The *ORF1* length differences observed were due to an insertion of three nt between positions 150–151 in the Spanish strains LE21006, LE21008, LE21016, LE21018 and LE21033 (Fig. 2a). This insertion translates into a proline (P) in the non-structural protein p16 (Fig. 2b), similar to the HaCV French strain E15-431 (GenBank accession number: MH204883)^20^. The Australian strain HaCV-A1/AUS/VIC/JM-29/2017 (GenBank accession number: MK138383) also presents a similar insertion^19^.

### Recombination analysis

Recombination analysis detected one recombination breakpoint (Table 1). The recombination breakpoint was located within the encoded non-structural protein 2C-like RNA helicase at nucleotide position 1889 (99% CI: 1864–1907). This breakpoint was detected in the Spanish strains LE21006, LE21008, LE21016, LE21018 and LE21033 and the most likely donor for the 3' fragment is LE20013 (2020, Spain; GII.1), while for the 5’ fragment the putative donor strain could not be identified. RDP also detected the HaCV French (strain E15-431; GenBank accession number: MH204883) and Italian (strain Bs12_1; GenBank accession number: KR230102) strains as potential EBHSV-HaCV recombinants (data not shown), but with a recombination breakpoint close to the RdRp/VP60 boundary.

**Table 1:** Recombination detection results using RDP.

Strains	Recombination breakpoint (nucleotide positions)	Most likely donor strain		Methods and average p-value						
		5′ to the recombination breakpoint	3′ to the recombination breakpoint	RDP	GENECONV	BootScan	MaxChi	Chimaera	SiScan	3Seq
LE21006 LE21008 LE21016 LE21018 LE21033	1889 (99% CI: 1864–1907)	Unknown	LE20013 2020 Spain (GII.1)	1.944 × 10^–05^	3.895 × 10^–04^	3.653 × 10^–06^	3.331 × 10^–06^	6.126 × 10^–10^	9.162 × 10^–40^	4.618 × 10^–18^

### Phylogenetic reconstruction

**Figure 3:**
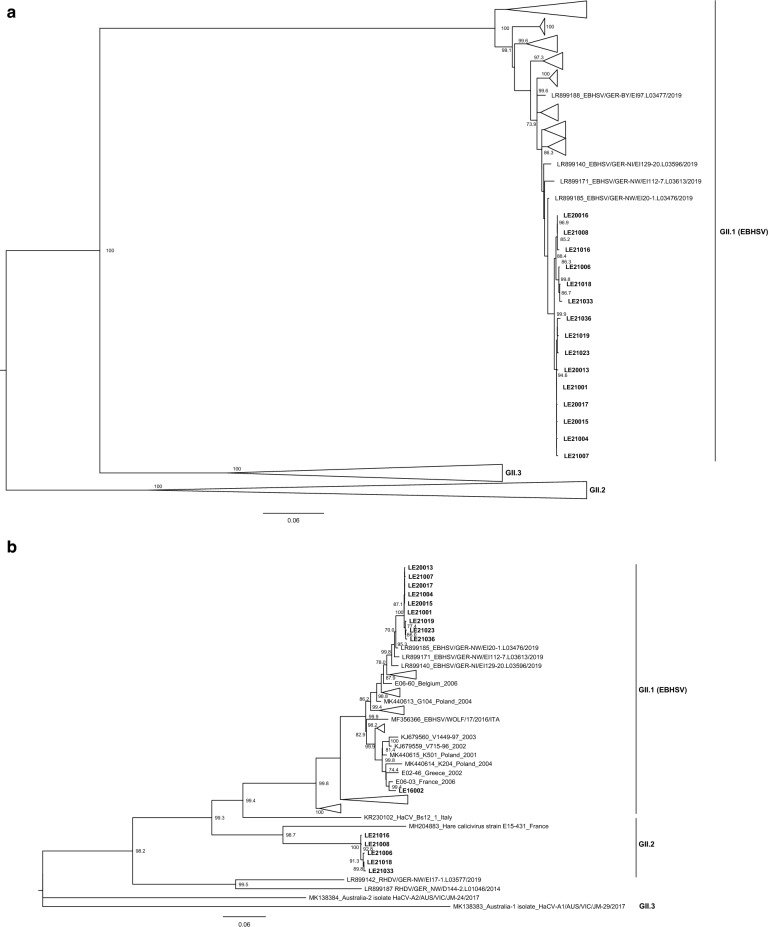

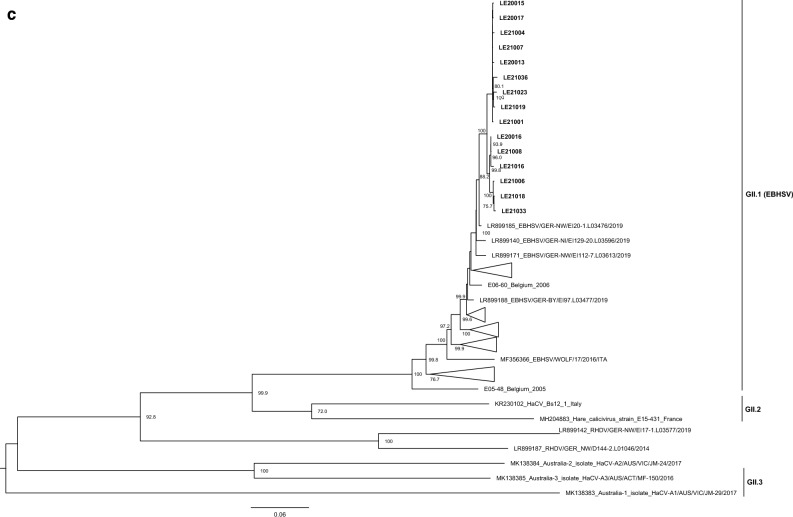
Phylogenetic analysis of the different genomic partitions. Maximum-likelihood (ML) phylogenetic trees were inferred for the (**a**) capsid gene (N = 173; 1728 nt) for genogroup and genotype assignment, according to the nomenclature proposed by^1^; (**b**) genomic fragment between nt positions 1–1889 (N = 67; 1889 nt); and (**c**) genomic fragment between nt positions 1890–5277 (N = 69; 3388 nt). GenBank accession numbers of sequences used are listed in Supplementary Tables 3 and 4. Horizontal branch lengths are drawn to scale of nucleotide substitutions per site. Trees were mid-point rooted and branch support was obtained from 1000 bootstrap replicates; only values above 70 are shown. The GTR + G + I model of nucleotide substitution was used for each tree. For clarity, some groups were collapsed.

Maximum-likelihood (ML) trees were inferred according to the recombination breakpoint detected, but also for the region encoding the capsid protein (nt positions 5278–7020; Fig. 3a) for genogroup and genotype assignment. In this tree (Fig. 3a), the sequences obtained in this study associated with the 2020/2021 outbreaks in Spain clustered together within a strongly supported group of GII.1 strains (bootstrap value of 100) and, thus, are GII.1 strains^1^. The strain LE16002 grouped with other European GII.1 strains (data not shown). Previously identified HaCV strains from Australia, France and Italy appear in two highly supported groups (bootstrap values of 100): one composed by Australian HaCV-A1 and A3 strains and a French HaCV strain (E15-226), which seem to correspond to GII.3^22^, and the other formed by the remaining HaCV strains, including the Australian HaCV-A2 (unassigned), the French E15-431 and the Italian Bs12_1 strains, which are assigned to GII.2^22^. For the ML tree corresponding to nucleotide positions 1–1889 (Fig. 3b), the Spanish strains appeared in two distinct clusters. Indeed, strains LE21006, LE21008, LE21016, LE21018 and LE21033 grouped with the French HaCV E15-431 strain (bootstrap value of 98.7). This group is closely related to a major group composed of GII.1 (EBHSV) strains, in which the remaining Spanish strains are included (non-recombinant GII.1), and the Italian HaCV Bs12_1 strain, which occupies a basal position (bootstrap value 99.4). The GII.1P-GI.2 strains^31^ appear as a strongly supported sister group (bootstrap value of 99.5), while the Australian HaCV-A2 and A1 strains are the most basal sequences in the tree. For the ML tree inferred for the fragment between nucleotide positions 1890–5277 (Fig. 3c), all the Spanish strains appear within the strongly supported GII.1 group (bootstrap value of 100). The Italian and French HaCV strains are basal to this group (bootstrap value of 72.0) and they group with strong statistically support with GII.1 (bootstrap value of 99.9). The GII.1P-GI.2 strains appear together, followed by the Australian HaCV-A2 and A3, which also cluster together (bootstrap values of 100). The Australian HaCV-A1 is the most basal sequence.

### Genetic distances estimation

Genetic distances (Table 2) showed that for the genomic fragment comprising nt positions 1–1889, the Spanish strains LE21006, LE21008, LE21016, LE21018 and LE21033 were more closely related to the French HaCV strain (0.162 ± 0.008). Distances to the other 2016–2021 Spanish strains (LE16002, LE20013, LE20015, LE20016, LE20017, LE21001, LE21004, LE21007, LE21019, LE21023 and LE21036) were higher (0.179 ± 0.008), with these strains presenting the lowest divergence to other GII.1 strains (0.070 ± 0.003). For the fragment between nt positions 1890–5277 (Table 3), genetic distances revealed that the Spanish strains LE21006, LE21008, LE21016, LE21018 and LE21033 were more similar to the 2016–2021 Spanish strains (0.018 ± 0.002). Distance to other GII.1 strains was 0.067 ± 0.003, while the distances to HaCV strains were much higher (between 0.191 ± 0.007 and 0.251 ± 0.007). For the capsid (nt positions 5278–7008), the Spanish strains LE21006, LE21008, LE21016, LE21018 and LE21033 were more closely related to the 2016–2021 Spanish strains (0.012 ± 0.004), followed by the other GII.1 strains (0.066 ± 0.004); distances to HaCV strains were between 0.235 ± 0.010 and 0.272 ± 0.009 (strains HaCV-A1 and E15-431, respectively) (see Supplementary Table 1).

**Table 2:** Genetic distances of the different groups in the region comprising nt 1–1889.

	SP_rec	SP_2016-2021 (GII.1)	GII.1	HaCV_FR (E15-431; GII.2)	HaCV_IT (Bs12_1; GII.2)	HaCV-A1 (GII.3)	HaCV-A2 (unassigned)
SP_rec		*0.008*	*0.007*	*0.008*	*0.008*	*0.010*	*0.009*
SP_2016-2021 (GII.1)	0.179		*0.003*	*0.009*	*0.008*	*0.010*	*0.009*
GII.1	0.185	0.070		*0.008*	*0.007*	*0.009*	*0.008*
HaCV_FR (E15-431; GII.2)	0.162	0.206	0.203		*0.008*	*0.009*	*0.009*
HaCV_IT (Bs12_1; GII.2)	0.184	0.174	0.173	0.203		*0.010*	*0.009*
HaCV-A1 (GII.3)	0.265	0.273	0.272	0.264	0.272		*0.010*
HaCV-A2 (unassigned)	0.235	0.238	0.240	0.255	0.233	0.259	

The genetic distances are shown as the number of base differences per site from averaging over all sequence pairs between groups. Standard error estimates are shown in italics.

SP_rec: Spanish HaCV-GII.1 recombinant strains; SP_ 2016–2021 (GII.1): Spanish non-recombinant GII.1 (EBHSV) strains collected in 2016 and between 2020 and 2021; GII.1: other EBHSV non-recombinant strains.

**Table 3:** Genetic distances of the different groups in the region comprising nt 1890–5277.

	SP_rec	SP_2016-2021 (GII.1)	GII.1	HaCV_FR (E15-431; GII.2)	HaCV_IT (Bs12_1; GII.2)	HaCV-A1 (GII.3)	HaCV-A2 (unassigned)	HaCV-A3 (GII.3)
SP_rec		*0.002*	*0.003*	*0.008*	*0.007*	*0.007*	*0.007*	*0.008*
SP_2016-2021 (GII.1)	0.018		*0.003*	*0.008*	*0.007*	*0.007*	*0.007*	*0.008*
GII.1	0.067	0.068		*0.007*	*0.006*	*0.007*	*0.006*	*0.007*
HaCV_FR (E15-431; GII.2)	0.196	0.197	0.195		*0.007*	*0.008*	*0.007*	*0.007*
HaCV_IT (Bs12_1; GII.2)	0.191	0.191	0.189	0.183		*0.007*	*0.007*	*0.007*
HaCV-A1 (GII.3)	0.251	0.251	0.249	0.243	0.241		*0.008*	*0.008*
HaCV-A2 (unassigned)	0.242	0.242	0.243	0.240	0.229	0.258		*0.008*
HaCV-A3 (GII.3)	0.241	0.245	0.241	0.245	0.238	0.261	0.205	

The genetic distances are shown as the number of base differences per site from averaging over all sequence pairs between groups. Standard error estimates are shown in italics.

SP_rec: Spanish HaCV-GII.1 recombinant strains; SP_ 2016–2021 (GII.1): Spanish non-recombinant GII.1 (EBHSV) strains collected in 2016 and between 2020 and 2021; GII.1: other EBHSV non-recombinant strains.

## Discussion

Our results support that the Spanish strains LE21006, LE21008, LE21016, LE21018 and LE21033 are the result of a recombination event involving an HaCV-like strain (yet to be classified from its capsid sequence according to^1^) and GII.1 (EBHSV), with a recombination breakpoint within the coding region of the non-structural protein 2C-like RNA helicase. This contrasts to the recombination hotspot observed to date in lagoviruses, which is located at the junction between the non-structural and structural encoding regions^19,23– 32^. In the reported recombinant strains, the fragment upstream of the recombination breakpoint is 83.8% similar to the non-pathogenic European hare calicivirus E15-431 (GII.2), while its similarity to GII.1 strains from the same outbreaks in Spain is 82.2%. Although these are not significantly different, we hypothesize that the 5′ fragment originated from a non-pathogenic rather than a pathogenic strain. Indeed, no closely related lagovirus sequences have been detected from hares found dead in the field, despite lagomorphs’ surveillance programs running in Catalonia since 2006^18^, and no antibodies against EBHSV have been detected earlier than 2016^18^. Thus, these strains constitute a novel type of recombinant; moreover, to our knowledge, they further correspond to the first report of a recombination event involving GII non-pathogenic and pathogenic strains. In addition, because these recombinant strains were retrieved from liver samples, the results suggest that both non-pathogenic and pathogenic EBHSV-related strains can replicate in the same target organ, the liver, as has been suggested from other recombination events involving pathogenic and non-pathogenic lagoviruses e.g.^32^. Our results also add to the growing body of evidence that recombination between non-pathogenic and pathogenic lagoviruses is quite frequent and can render them lethal to their hosts or even novel hosts e.g.^26,27^.

The “atypical” recombination breakpoint indicates that non-structural proteins p16, p23 and part of the 2C-like RNA helicase originated from an unknown non-pathogenic strain. Studies aimed at determining the role of such proteins are restricted to RHDV, but the close evolutionary relationship between RHDV and EBHSV suggests that their proteins might share similar biological functions. While the RHDV structural proteins seem to be associated with host and tissue tropism, which is linked with pathogenicity^32^, non-structural proteins with known function are implicated in virus replication (RdRp, helicase and VPg), translation (VPg) and viral polyprotein processing (protease)^36–41^. The other non-structural proteins p16, p23 and p29 seem to be also involved in viral replication and translation, and a role in downregulation of host immune responses has also been suggested^42^. The RHDV p16 protein accumulates in subnuclear compartments and an interaction with nucleic acids and/or cellular proteins has been initially proposed^43^. More recently, Zhu and co-workers^44^ observed an interaction of host nucleolin (NCL) with p16, which led to the speculation that RHDV might take over the NCL-associated machinery of rRNA transcription and pre-rRNA processing to replicate its gRNA. As for the p23 protein, it is localized to the endoplasmic reticulum (ER) where it oligomerizes and acts as a viroporin by forming ion channels in the membranes^43,45^. In other caliciviruses, viroporins trigger apoptosis, which likely contributes to virus spreading^42^. NCL also interacts with p23, probably for the recruitment of host proteins associated with replication into the ER membrane to form replication complexes^44^. Therefore, since p16, p23 and helicase seem to have a crucial role in virus replication and spreading, it remains to determine if their non-pathogenic origin might interfere with such processes in the recombinant strains.

The earliest evidence of the circulation of the new recombinant dates from February 2021 from a juvenile dead hare collected in Pla de L’Estany, Girona, Spain. From this location, the recombinant strains were detected in regions 20–100 km apart in Catalonia, Spain, in different seasons. For example, strain LE21033 was isolated from a juvenile hare found dead in Anoia, Barcelona, in June 2021, while the remaining were isolated from dead adult hares from two other locations in Girona (two in Selva and one in Ripollès), which were collected in February-April 2021. This shows that these recombinant strains managed to spread into different hare populations in Catalonia, and were able to be transmitted and establish infection not only in juvenile, but also in adult hares. This suggests that, despite their “atypical” recombination breakpoint, the recombinant strains might present some epidemiological relevance. However, their close similarity to the contemporary non-recombinant GII.1 strains suggests a recent origin in Catalonian hare populations and, thus, a continuous monitoring in the following years is needed to understand if the recombinant will thrive in the hare populations.

This study constitutes the first molecular characterization of non-pathogenic EBHSV-related (HaCV) strains in the Iberian Peninsula, even if only a partial sequence was obtained. Their circulation in Iberian European brown hares had been previously suggested^18^, and this work provides further evidence for their circulation. We speculate that the prevalence of this non-pathogenic strain in the Spanish hare populations might be rather high, increasing the chances of recombination. The genetic distances estimated for the partial sequence obtained for the non-structural region (nt positions 1–1889) indicate, as observed and pointed out for the complete coding genome sequences of other HaCV strains^19–22^, a high divergence within this group, which might be due to their long-time circulation within hare populations. Such divergence (in some instances, higher than 27%) might suggest the need to add more genogroups other than GI and GII, which differ ~ 30% (nt), to the *Lagovirus europaeus* nomenclature. Indeed, when the nomenclature by Le Pendu et al.^1^ was proposed, the capsid genetic distance to define new genogroups was not considered because no such high diversity had been reported. This should now be re-evaluated. Furthermore, these results also reinforce our limited knowledge on the diversity of non-pathogenic lagoviruses circulating in leporids and the need for more in-depth studies, particularly because of their role in the emergence and evolution of these viruses, as has been demonstrated for GI.1 and GI.2, e.g.^23,26,35^. Finally, since these recombinant strains seemed to be associated with mortality in European hares, close monitoring of the impact of this novel HaCV-EBHSV recombinant in hare populations in the Iberian Peninsula is advised.

## Methods

### Necropsy and sample collection

Between December 2020 and July 2021, unusual mortalities occurred in European brown hare populations from Catalonia, Spain. Hares (n = 40) were collected by hunters and Rural Agents and transported to the Veterinary Faculty of the Universitat Autònoma de Barcelona, Spain. A systematic necropsy and histopathological investigation were performed under the Passive Surveillance Program for game species in the region (https://agricultura.gencat.cat/ca/ambits/ramaderia/sanitat-animal/pla-vigilancia-sanitaria-fauna-salvatge/). As part of the routine, liver (n = 39) and bone marrow (n = 1) samples were collected from all animals during the necropsy and preserved at -20 ºC. Twenty-five animals presented macroscopic and microscopic lesions consistent with lethal lagovirus infection, such as periportal to massive coagulation hepatocellular necrosis^4^. For the remaining animals, causes of death included bacterial septicaemia, bacterial bronchopneumonia, bacterial dermatitis, pseudotuberculosis, and trauma. The collected liver and bone marrow samples were further analyzed by biomolecular assays for lagovirus confirmation and characterization. Additionally, a liver sample from an European brown hare found dead in 2016 with EBHS-compatible lesions and belonging to the last EBHSV outbreak reported in the same area was incorporated in this study. Lagovirus-positive hares were identified as European brown hares (*Lepus europaeus*) according to phenotypic morphometric characters^46^, and further classified into males (N = 5), females (N = 11), and adults (N = 13) and juveniles (N = 3) by studying radium-ulna ossification^47^. Data regarding their origin, sampling date, sex, weight, and age are presented in Table 4. Ethical approval was not sought or required as no live animals were used and all samples were recovered from animals that died from natural causes, and diagnostic procedures were performed as part of standard pathological investigations. All sampling and handling procedures were conducted by authorized personnel.

**Table 4:** List of the lagovirus-positive samples and related information.

Sample ID	Species	Location			Collection date (dd/mm/yy)	Age	Weight (kg)	Sex
		City	Region	Province				
LE16002	*Lepus europaeus*	Navata	Alt Emporda	Girona	01/02/16	Adult	3.18	M
LE20013		Les Avellanes i Santa Linya	Noguera	Lleida	05/12/20	Juvenile	3.28	F
LE20015		Sant Marti de Tous	Anoia	Barcelona	14/12/20	Adult	3.78	F
LE20016		Cassa de la Selva	Girones	Girona	17/12/20		3.32	F
LE20017		Vall de Boi	Alta Ribagorça	Lleida	18/12/20		2.90	F
LE21001		Calonge de Segarra	Anoia	Barcelona	04/01/21		3.70	M
LE21004		Pontils	Conca de Barbera	Tarragona	03/01/21		4.18	F
LE21006*		Serinya	Pla de l’Estany	Girona	04/02/21	Juvenile	3.02	M
LE21007		Montmell	Baix Penedes	Tarragona	06/02/21	Adult	3.58	M
LE21008*		Amer	Selva	Girona	14/02/21		3.84	F
LE21016*		Sant Feliu de Buixalleu	Selva	Girona	22/03/21		1.64**	F
LE21018*		Molló	Ripolles	Girona	06/04/21		3.56	F***
LE21019		Ribes de Freser	Ripolles	Girona	07/04/21		4.14	F
LE21023		Planoles	Ripolles	Girona	19/04/21		2.70	M
LE21033*		La Molsosa	Anoia	Barcelona	22/06/21	Juvenile	1.90	F
LE21036		Les Valls d'Aguilar	Alt Urgell	Lleida	27/04/21	Adult	3.88	F

*Recombinant strain; **without viscera; ***pregnant: one leveret.

### Viral RNA extraction, cDNA synthesis and sequencing

A portion of liver sample (approximately, 30 mg) from each hare was homogenized in a rotor–stator homogenizer (Mixer Mill MM400, Retsch) at 30 Hz for 5 min. Total RNA was extracted using the GeneJET RNA Purification kit (Thermo Scientific) and viral cDNA was synthesized with the NZY first-strand cDNA synthesis kit (Nzytech), both according to the manufacturers’ protocols. For sample LE21016, no viscera was recovered at the time of necropsy and RNA was extracted from bone marrow.

Screening for lagoviruses was performed in two rounds of PCR, one for GI.2 detection and the other for GII.1 detection (Supplementary Table 2). Both PCRs used the Phusion Flash High-Fidelity PCR Master Mix (Thermo Scientific), 2 pmol of each primer, 1 μL of cDNA and ultra-pure water for a final PCR volume of 10 μL. Cycle conditions were: 98 °C for 3 min, followed by 40 cycles of 30 s at 98 °C, 30 s at the annealing temperature and extension at 72 °C; a final extension step of 5 min at 72 °C terminated the PCR reaction. PCR primer sequences, annealing temperatures and extension times are indicated in Supplementary Table 2. PCR positive results (i.e., amplification products with the correct expected size) were purified and sequenced by Sanger sequencing on an automatic sequencer ABI PRISM 3500XL Genetic Analyzer (PE Applied Biosystems) using the amplification primers.

A primer-walking strategy was used to obtain complete coding sequences as described elsewhere^48^. Briefly, overlapping genomic fragments were obtained by PCR using primers designed in conserved regions of the EBHSV genome (Supplementary Table 2); new primers were further designed in conserved regions of HaCV genomes to complete missing information in the sequences. Sequencing was as described above. The sequences were deposited in GenBank and the accession numbers are included in the Supplementary Tables 3 and 4.

### Detection of recombination, estimation of genetic distances and phylogenetic reconstruction

The sequences obtained were aligned using BioEdit version 7.0.3^49^ with complete coding sequences of GII.1 and HaCV strains and the previously reported GII.1P-GI.2 recombinant strains^31^ available at GenBank. The final dataset consisted of 69 sequences, 7357 nucleotides in length (see Supplementary Table 4 for the list of the sequences used).

The alignment was screened for recombination using the Recombination Detection Program 5 (RDP5)^50^ with the following parameters: linear sequences, Bonferroni correction, and highest acceptable p-value = 0.05. Only recombination events detected by at least three methods (p < 0.05) were considered.

Following the results obtained with RDP, genetic distances were calculated in MEGA11^51^ for each of the regions 5’ and 3’ to the recombination breakpoints to find the highest similarities to the putative recombinant strains. The following parameters were used: p-distance, partial deletion (95%) and 1000 bootstrap replicates.

For the non-structural part, maximum-likelihood (ML) phylogenetic trees were inferred separately for each genomic partition using MEGA11^51^. The GTR + G + I model of nucleotide substitution was used, as determined in the same software and according to the lowest AICc value (Akaike information criterion, corrected). Branch support was obtained from 1000 bootstrap replicates. In the analysis focusing on the genomic fragment between nt 1–1889, the Australian HaCV-A3 strain (GenBank accession number: MK138385) and the Spanish strain LE20016 were excluded as the nucleotide sequences were incomplete for this region. A ML tree was also constructed for the capsid gene (nt positions 5278–7008) for genogroup and genotype assignment^1^ using the same options described above and including all EBHSV and HaCV capsid sequences available in public databases (N = 173; see Supplementary Table 3 for the list of the sequences used).

### Supplementary Information

Supplementary Tables.

## Data Availability

All sequences generated in this study were deposited in the GenBank database (https://www.ncbi.nlm.nih.gov/nuccore/) under the following accession numbers: OQ674058–OQ674073.
