# Supplementary material for: A new HaCV-EBHSV recombinant lagovirus circulating in European brown hares (Lepus europaeus) from Catalonia, Spain
Source: Sci Rep. 2024 Feb 4;14:2872. doi: 10.1038/s41598-024-53201-1 (PMC10838927; doi:10.1038/s41598-024-53201-1)
Supplement: Supplementary file 1 — Supplementary Tables. [file 41598_2024_53201_MOESM1_ESM.pdf]

A new HaCV-EBHSV recombinant lagovirus circulating in European brown hares (*Lepus europaeus*) from Catalonia, Spain

Tereza Almeida, Ana M. Lopes, Josep Estruch, Carlos Rouco, Patrizia Cavadini, Aleksija Neimanis, Dolores Gavier-Widén, Ghislaine Le Gall-Reculé, Roser Velarde, Joana Abrantes

Supplementary Information

Supplementary Table 1. Genetic distances of the different groups in the region encoding the capsid gene (5278-7008). The genetic distances are shown as the number of base differences per site from averaging over all sequence pairs between groups. Standard error estimates are shown in italics

	SP_rec	SP_2016-2021 (GII.1)	EBHSV (GII.1)	HaCV (GII.2)	HaCV_FR (E15-431; GII.2)	HaCV_IT (Bs12_1; GII.2)	HaCV-A1 (GII.3)	HaCV-A2 (unassigned)	HaCV-A3 (GII.3)	HaCV_FR (E-15-226; unassigned)
SP_rec		*0.002*	*0.004*	*0.007*	*0.009*	*0.009*	*0.009*	*0.009*	*0.010*	*0.009*
SP_2016-2021 (GII.1)	**0.012**		*0.004*	*0.007*	*0.009*	*0.009*	*0.009*	*0.009*	*0.010*	*0.009*
EBHSV (GII.1)	0.066	0.065		*0.007*	*0.009*	*0.008*	*0.008*	*0.009*	*0.009*	*0.009*
HaCV (GII.2)	0.267	0.265	0.267		*0.005*	*0.004*	*0.007*	*0.006*	*0.008*	*0.007*
HaCV_FR (E15-431; GII.2)	0.272	0.273	0.268	0.162		*0.007*	*0.009*	*0.009*	*0.010*	*0.010*
HaCV_IT (Bs12_1; GII.2)	0.268	0.265	0.271	0.136	0.153		*0.009*	*0.009*	*0.010*	*0.009*
HaCV-A1 (GII.3)	0.235	0.234	0.241	0.258	0.252	0.254		*0.009*	*0.009*	*0.008*
HaCV-A2 (unassigned)	0.271	0.270	0.270	0.219	0.222	0.215	0.243		*0.010*	*0.009*
HaCV-A3 (GII.3)	0.241	0.244	0.244	0.262	0.255	0.264	0.180	0.250		*0.008*
HaCV_FR (E15-226; unassigned)	0.246	0.245	0.43	0.261	0.253	0.257	0.199	0.260	0.202	

SP_rec: Spanish HaCV/EBHSV recombinant strains; SP_ 2016-2021 (GII.1): Spanish non-recombinant EBHSV strains collected in 2016 and between 2020-2021; GII.1: other EBHSV non-recombinant strains

Supplementary Table 2. PCR primers and amplification conditions.

Primer name	Sequence (5'- 3')	Annealing temperature (ºC)	Extension time	Product length (region covered***)	Reference
RHDV6186F*<br>RHDV6748R*	CATTGACCACGACAGAGGTAAC<br>CGTTAGTTGAACCGGCCTCAG	67	20''	562 bp (6143-6705)	Rouco et al. (2018)
U38F**<br>EBHS9R**	CAGCGGGCACTGCTACCACAGCATC<br>CCAGCCCAACCAGCRTACAT	55	15''	307 bp (5299-5605)	Lopes et al. (2013)
EBHSV0001F<br>EBHSV1085R	GTGAAATTATGGCGGTTGCG<br>GGACTGAGTCCTCAAACTTG	55	50''	1104 bp (-8-1095)	
EBHSV0001F<br>EBHSV0504R	GTGAAATTATGGCGGTTGCG<br>CAAGRTCAACAAACCTGTCCATG	55	20''	534 bp (-8-525)	
EBHSV0908F<br>EBHSV1977R	CCTGCTGGATTGGACAAATGAC<br>GAAGGTGAGGTGAGACATGTC	54	50''	1090 bp (899-1988)	
EBHSV1761F<br>EBHSV2745R	CCTCTCAACTGTGACAAGGTTG<br>GGTCACCAGAGTATCTCACAAG	49	1'15''	1006 bp (1752-2757)	
EBHSV2615F<br>EBHSV3835R	CAATCCGGTGTGTGCATATG<br>CCTGGAACCTTTGCATACCTTG	49	1'15''	1242 bp (2606-3847)	
EBHSV3767F<br>EBHSV4604R	CATTGACTACCGTGGACTTG<br>GAACGGTCATTGGAAGTGAC	51	1'15''	857 bp (3758-4614)	
EBHSV4503F<br>EBHSV5320R	GATTCCACGATGTCACCATG<br>AGGAACAGATGCTGTGGTAG	54	50''	837 bp (4494-5330)	
EBHSV_VP60_0001F<br>EBHSV_VP60_1728R	ATGGAGGGTAAGCCWCGGGCTGA<br>GACATAGGAATATCCAGTGGTGGC	54	1'30''	1728 bp (5274-7001)	
EBHSV6911F<br>EBHSV7337R	GACAGACCTCATTGACGTG<br>CAAAYCGCTAGGCGTTACTC	54	30''	447 bp (6902-7348)	
HaCV379F<br>HaCV1128R	CCTTGCCTGTTCACTCGATC<br>CAACTGCTGGCTGTCTGATC	60	40''	750 bp (375-1124)	This study
HaCV414F<br>HaCV1526R	CGAAGGTGAAGTTGAAGACC<br>CAACATGACAAAACAGCGACC	60	40''	1113 bp (410-1522)	
HaCV1346F<br>HaCV2368R	CAACCCTCAGCCAACTTGAG<br>CAAAGTGTGGGACCATCCTC	62	30''	1023 bp (1342-2364)	
HaCV1410F<br>HaCV2414R	CAGATGCACACTTCCCTCCT<br>GTCAAGTCGGTGCCTAATTGG	62	30''	1005 bp (1406-2410)	

*GI.2 detection; **GII.1 detection; ***according to reference sequence EBHSV-GD (GenBank accession number: Z69620)

Rouco, C. et al. Epidemiology of RHDV2 (Lagovirus europaeus/GI.2) in free-living wild European rabbits in Portugal. Transbound. Emerg. Dis. 65, e373-e382, doi:10.1111/tbed.12767 (2018).

Lopes, A. M., Gavier-Widen, D., Le Gall-Reculé, G., Esteves, P. J. & Abrantes, J. Complete coding sequences of European brown hare syndrome virus (EBHSV) strains isolated in 1982 in Sweden. Arch. Virol. 158, 2193-2196, doi:10.1007/s00705-013-1714-7 (2013).

Supplementary Table 3. List of the VP60 sequences used.

GenBank Accession Number	Strain
AJ971301	EBHSV_i0252_G1-1_France_2002
AJ971304	E0251_France_2002
AJ971305	E01-01_France_2001
AJ971306	E02-56_France_2002
AJ971311	EBHSV i0102 G2-2_France_2001
AJ971315	EBHSV i0305 G2-3_France_2003
AM408588	E0330_France_2003
AM887765	E0540_France_2005
AM933648	EBHSV_s0603 G2-3_France_2006
AM933649	EBHSV_s0636 G3_France_2006
AM933650	EBHSV_s0516 G3_France_2005
FN689419	E03-23_France_2003
FN689420	E04-35_France_2004
FN689421	E04-53_France_2004
HF571039	E0822_France_2008
HF571040	E0836_France_2008
JX195102	MI09_Italy_2009
KF591083	MI09_1_Italy_2009
KJ679513	O484_Sweden_1982
KJ679514	O3891_Sweden_1982
KJ679515	O4021_Sweden_1982
KJ679516	O4022_Sweden_1982
KJ679517	O297_Sweden_1983
KJ679518	O575_Sweden_1983

KJ679519	O591_Sweden_1983
KJ679520	O3303_Sweden_1983
KJ679521	O3305_Sweden_1983
KJ679522	O282_Sweden_1984
KJ679523	O1000_Sweden_1984
KJ679524	O1183_Sweden_1984
KJ679525	O2159_Sweden_1984
KJ679526	O2485_Sweden_1984
KJ679527	O3023_Sweden_1984
KJ679528	O3024_Sweden_1984
KJ679529	V25_Sweden_1985
KJ679530	V78_Sweden_1985
KJ679531	V1223_Sweden_1987
KJ679532	V1645_Sweden_1987
KJ679533	V1836_Sweden_1987
KJ679534	V1846_Sweden_1987
KJ679535	V1862_Sweden_1987
KJ679536	V1869_Sweden_1987
KJ679537	V1900_Sweden_1987
KJ679538	V1970_Sweden_1987
KJ679539	V1974_Sweden_1987
KJ679540	V392_Sweden_1988
KJ679541	V1220_Sweden_1988
KJ679542	V1278_Sweden_1988
KJ679543	V1392_Sweden_1988
KJ679544	V1394_Sweden_1988

KJ679545	V1437_Sweden_1988
KJ679546	V1466_Sweden_1988
KJ679547	V1519_Sweden_1988
KJ679548	V1571_Sweden_1988
KJ679549	V954_Sweden_1989
KJ679550	V871_Sweden_1993
KJ679551	V912_Sweden_1993
KJ679552	V1166_Sweden_1993
KJ679553	V58_Sweden_1994
KJ679554	V961_Sweden_1994
KJ679555	V212_Sweden_1996
KJ679556	V24_Sweden_1998
KJ679557	V117_Sweden_1998
KJ679558	V171_Sweden_2001
KJ679559	V715_Sweden_2002
KJ679560	V1449_Sweden_2003
KJ679561	V707_Sweden_2008
KJ679562	V708_Sweden_2008
KJ679563	V1069_Sweden_2008
KJ679564	V1070_Sweden_2008
KJ679565	V1703_Sweden_2008
KJ679566	V2070_Sweden_2008
KJ923230	EBHSV_Italy_1992
KU961677	Bs15_1
KU961678	Bs15_2
KY801206	B-EBHS-6

MF356366	EBHSV/WOLF/17/2016/ITA
U09199	EBHSV_Germany_1989
X98002	EBHSV_BS89_Italy_1989
Z32526	EBHSV-GD
Z69620	EBHSV_France_1989
LR899140	EBHSV/GER-NI/EI129-20.L03596/2019
LR899152	EBHSV/GER-TH/EI04-2.L03594/2020
LR899171	EBHSV/GER-NW/EI112-7.L03613/2019
LR899182	EBHSV/GER-BY/EI104-12.L03475/2019
LR899185	EBHSV/GER-NW/EI20-1.L03476/2019
LR899188	EBHSV/GER-BY/EI97.L03477/2019
MK440613	G104
MK440614	K204
MK440615	K501
MK440616	L98
MK440617	NP1192
LT168848	E14-40/2
MG781002	HaCV_Lu16
MG781003	HaCV_Lu15
MG781004	HaCV_Bs10
MG781005	HaCV_So14
MG781006	HaCV_So14_2
MG781007	HaCV_GH11
MG781008	HaCV_PE11
MG781009	HaCV_UD11
MN128592	HaCV_E15-377

MH992067	HaCV_E15-428
MH992068	HaCV_E15-425
MH992069	HaCV_E15-384
MH992070	HaCV_E15-374
MH992071	HaCV_E15-338
MH992072	HaCV_E15-304
MH992073	HaCV_E15-226
MH992074	HaCV_E15-219
MH992075	HaCV_E14-73b
KR230103	HaCV_Bs12_2
KR230104	HaCV_Bs14_1
KR230105	HaCV_Bs14_2
KR230106	HaCV_Bs14_3
KR349359	HaCV_Pc14
KR349360	HaCV_Mo14
KR349361	HaCV_Re14_1
KR349362	HaCV_Re14_2
KT943469	HaCV Bs15
KT985456	HaCV_Bs15_1
MK138383	HaCV-A1/AUS/VIC/JM-29/2017
MK138384	HaCV-A2/AUS/VIC/JM-24/2017
MK138385	HaCV-A3/AUS/ACT/MF-150/2016
MH204883	HaCV_E15-431
KR230102	HaCV_Bs12_1
AJ584643	E02-46_Greece_2002
AM933648	E06-03_France_2006

AM933649	E06-36_France_2006
HG329733	E06-60_Belgium_2006
HG329732	E05-48_Belgium_2005
HG329734	E09-45_Belgium_2009
OQ674062	LE20013
OQ674063	LE20015
OQ674071	LE20016
OQ674064	LE20017
OQ674065	LE21001
OQ674066	LE21004
OQ674071	LE21016
OQ674061	LE16002
OQ674069	LE21006
OQ674070	LE21008
OQ674072	LE21018
OQ674059	LE21023
OQ674068	LE21007
OQ674073	LE21033
OQ674060	LE21036
OQ674067	LE21019
KJ679526	O2485-28_Sweden_1984
KJ679522	O282-23_Sweden_1984
KJ679513	O484-1_Sweden_1982
KJ679519	O591-16_Sweden_1983
OQ674051	Sylvilagus_Italy
KJ679564	V1070-101_Sweden_2008

KJ679552	V1166-80_Sweden_1993
KJ679557	V117-94_Sweden_1998
KJ679531	V1223-37_Sweden_1987
KJ679560	V1449-97_Sweden_2003
KJ679532	V1645-40_Sweden_1987
KJ679565	V1703-102_Sweden_2008
KJ679558	V171-95_Sweden_2001
KJ679538	V1970-54_Sweden_1987
KJ679555	V212-90_Sweden_1996
KJ679556	V24-93_Sweden_1998
KJ679553	V58-81_Sweden_1994
KJ679559	V715-96_Sweden_2002
KJ679554	V961-84_Sweden_1994
OQ674057	VLT000093_Sweden_2016
OQ674055	VLT000190_Sweden_2016
OQ674053	VLT000203_Sweden_2009
OQ674054	VLT000443_Sweden_2015
OQ674052	VLT000987_Sweden_2009
OQ674056	VLT001722_Sweden_2009

Supplementary Table 4. List of the complete coding sequences used.

**Accession number**	**Strain**
KC832838	European brown hare syndrome virus isolate O4022-10_1982
KC832839	European brown hare syndrome virus isolate O4021-9_1982
KR230102	HaCV_Bs12_1_Italy
LR899140	EBHSV/GER-NI/EI129-20.L03596/2019
LR899152	EBHSV/GER-TH/EI04-2.L03594/2020
LR899171	EBHSV/GER-NW/EI112-7.L03613/2019
LR899182	EBHSV/GER-BY/EI104-12.L03475/2019
LR899185	EBHSV/GER-NW/EI20-1.L03476/2019
LR899142	RHDV/GER-NW/EI17-1.L03577/2019
LR899187	RHDV/GER/NW/D144-2.L01046/2014
LR899188	EBHSV/GER-BY/EI97.L03477/2019
MF356366	EBHSV/WOLF/17/2016/ITA
MH204883	Hare calicivirus strain E15-431_France
MK138383	Australia-1 isolate_HaCV-A1/AUS/VIC/JM-29/2017
MK138384	Australia-2 isolate HaCV-A2/AUS/VIC/JM-24/2017
MK138385	Australia-3 isolate HaCV-A3/AUS/ACT/MF-150/2016
Z69620	European brown hare syndrome virus RNA
MK440613	G104_Poland_2004
MK440614	K204_Poland_2004
MK440615	K501_Poland_2001
MK440616	L98_Poland_1998
MK440617	NP1192_Poland_1992
AJ584643	E02-46_Greece_2002
AM933648	E06-03_France_2006

AM933649	E06-36_France_2006
HG329733	E06-60_Belgium_2006
HG329732	E05-48_Belgium_2005
HG329734	E09-45_Belgium_2009
OQ674062	LE20013
OQ674063	LE20015
OQ674071	LE20016
OQ674064	LE20017
OQ674065	LE21001
OQ674066	LE21004
OQ674071	LE21016
OQ674061	LE16002
OQ674069	LE21006
OQ674070	LE21008
OQ674072	LE21018
OQ674059	LE21023
OQ674068	LE21007
OQ674073	LE21033
OQ674060	LE21036
OQ674067	LE21019
KJ679526	O2485-28_Sweden_1984
KJ679522	O282-23_Sweden_1984
KJ679513	O484-1_Sweden_1982
KJ679519	O591-16_Sweden_1983
OQ674051	Sylvilagus_Italy
KJ679564	V1070-101_Sweden_2008

KJ679552	V1166-80_Sweden_1993
KJ679557	V117-94_Sweden_1998
KJ679531	V1223-37_Sweden_1987
KJ679560	V1449-97_Sweden_2003
KJ679532	V1645-40_Sweden_1987
KJ679565	V1703-102_Sweden_2008
KJ679558	V171-95_Sweden_2001
KJ679538	V1970-54_Sweden_1987
KJ679555	V212-90_Sweden_1996
KJ679556	V24-93_Sweden_1998
KJ679553	V58-81_Sweden_1994
KJ679559	V715-96_Sweden_2002
KJ679554	V961-84_Sweden_1994
OQ674057	VLT000093_Sweden_2016
OQ674055	VLT000190_Sweden_2016
OQ674053	VLT000203_Sweden_2009
OQ674054	VLT000443_Sweden_2015
OQ674052	VLT000987_Sweden_2009
OQ674056	VLT001722_Sweden_2009
